# A meta-analysis of childhood maltreatment in relation to psychopathic traits

**DOI:** 10.1371/journal.pone.0272704

**Published:** 2022-08-10

**Authors:** Corine de Ruiter, Matthias Burghart, Raneesha De Silva, Sara Griesbeck Garcia, Ushna Mian, Eoin Walshe, Veronika Zouharova

**Affiliations:** 1 Faculty of Psychology and Neuroscience, Maastricht University, Maastricht, the Netherlands; 2 Department of Clinical Psychology, University of Konstanz, Konstanz, Germany; University of Bern, SWITZERLAND

## Abstract

Psychopathy is a personality disorder characterized by a mix of traits belonging to four facets: affective (e.g., callous/lack of empathy), interpersonal (e.g., grandiosity), behavioral instability (e.g., impulsivity, poor behavioral controls), and social deviance (e.g., juvenile delinquency, criminal versatility). Several scholars have argued that early childhood maltreatment impacts the development of psychopathy, although views regarding its role in the four facets differ. We conducted a meta-analysis including 47 studies comprising a total of 389 effect sizes and 12,737 participants, to investigate the association between psychopathy and four types of child maltreatment: physical abuse, emotional abuse, neglect, and sexual abuse. We found support for a moderate link between overall psychopathy and childhood physical abuse, emotional abuse, and neglect, as well as overall childhood maltreatment. The link between psychopathy and childhood sexual abuse was small, but still statistically significant. These associations were stronger for the behavioral and antisocial facets than for the affective and interpersonal facets of psychopathy, but nearly all associations were statistically significant. Our findings are consistent with recently developed theories on the role of complex trauma in the development of severe personality disorders. Trauma-focused preventive and therapeutic interventions can provide further tests of the trauma-psychopathy hypothesis.

## Introduction

I had a kind of rough childhood. My Mom was admitted to a psychiatric institution many times, and my Dad was extremely harsh. He and my older brothers used to hit me with a broom. They also made me sit in a corner with hot peppers in my mouth. My memories of my childhood are rather chaotic, I do not like to think about that period. I was bullied in elementary school. But then, one day, I remember I hit this kid in the school yard during recess, and that’s when I found out I could be a bully too. And from that time on, I switched from being a victim to being the perpetrator. (Patient A.)

This quote is taken from a biographical interview administered in connection with the coding of the Psychopathy Checklist-Revised (PCL-R) for a 35-year old male violent offender admitted to a secure forensic psychiatric hospital. His criminal history is long and versatile; he assaulted and cheated on multiple intimate partners, embezzled money from a former employer, and he takes pride in his ability to manipulate others. He lacks remorse for any of his wrongdoings. He reports his family home environment was chaotic and he suffered maltreatment as a child, but he does not want to dwell on this. His consensus PCL-R score, based on the interview and extensive collateral information, amounted to 38, with 40 being the maximum score on the instrument.

Most contemporary conceptualizations of psychopathy are rooted in Cleckley’s clinically-derived symptom criteria [1]. Over the years, different theoretical and factorial models of the disorder have been developed [2–4], with a continuing debate pertaining to the question whether criminal/antisocial behavior should be included as part of the diagnostic criteria [5, 6]. Still, there is consensus in the field that psychopathy is a dimensional construct, consisting of affective, interpersonal and behavioral trait dimensions [7]. The terms to define these dimensions differ between the various models and psychopathy measures. For example, the triarchic model [8] uses the term ‘meanness’ to refer to the affective dimension, which is termed ‘callous-unemotional’ [9] or ‘deficient affective experience’ [10] in other models. Similarly, the interpersonal dimension is called ‘boldness’ in the triarchic model, but ‘narcissism’ [11] by others. The behavioral dimension has been termed ‘disinhibition’, ‘impulsivity’ and ‘lifestyle’.

Over the years, many different measures of these psychopathic trait dimensions have been designed and tested. These measures use different information sources, notably self-report, clinician judgment, and observer report. Prominent among these measures are the so-called Hare PCL scales: the Psychopathy Checklist-Revised (PCL-R) for adults, the Psychopathy Checklist: Youth Version (PCL:YV) for adolescents and the Antisocial Process Screening Device for children. The PCL-R (and its screening version, the PCL-SV) and the PCL:YV scales have a similar, 4-facet factor structure [12] consisting of an interpersonal, affective, lifestyle and antisocial facet. Originally, factor analyses of the PCL-scales had yielded two higher order factors (interpersonal/affective–Factor 1 and lifestyle/antisocial–Factor 2), which exhibit moderate to strong intercorrelations [13]. Currently, the 4-facet structure appears to be the dominant factorial model as it has been repeatedly supported by Confirmatory Factor Analysis and Structural Equation Modeling using different PCL-scales in samples of adolescents and adults, from community, offender and clinical settings, from North America and Europe (e.g., [14]; for an overview see [5]).

### Etiology of psychopathy: Theories about the role of childhood maltreatment

Speculating about the etiology of psychopathy, Cleckley [1] tended more towards an organic origin, although not necessarily hereditary: “If an inborn biologic defect exists and plays an important part in such a psychopath’s disorder, it is not necessary to assume that the defect is hereditary. Perhaps it may be the result of a subtle failure in maturation, an agenesis of unknown etiology” (p. 412). Checkley also showed appreciation for the work of Karpman [15] who maintained that psychogenic factors are responsible for the majority of cases of psychopathy. For a psychoanalyst like Karpman [16], ‘psychogenic’ largely referred to so-called oedipal conflicts, although he was also attuned to the relevance of parental maltreatment and rejection in the etiology of ‘secondary psychopathy’, as he termed it. This secondary psychopathy should be distinguished from primary psychopathy which, according to Karpman [16], was present in only a minority of cases (15%; p. 487) in which an inborn or constitutional defect caused the disorder.

Although most mental health problems are likely the result of complex interactions between ‘innate’ and ‘acquired’ components [17], over the past decades research into psychopathy has been dominated by a focus on its genetic and neurobiological underpinnings (e.g., [18–20]). Hare’s view that the disorder has a neurobiological and/or genetic origin and is therefore basically untreatable [21–23] had a notable impact on this. Recently, Frazier and colleagues [20] provided cogent criticism on this disbalance in the psychopathy research literature. They demonstrated that developmental research has consistently shown that children exposed to neglect, abuse, and other types of childhood maltreatment (CM) exhibit many of the same neurobiological abnormalities observed in adults with high scores on psychopathy [20]. A thorough review of this research literature is beyond the scope of the present paper. We will provide some recent examples from empirical studies that illustrate the complex nature of the association between CM and neurobiological functioning as it relates to the affective facet of psychopathy.

The affective facet of psychopathy has been shown to be related to a reduced psychophysiological response to negative emotional stimuli [24, 25] and suppression of neural activity and/or volume of the amygdalae [26–28], a limbic structure responsive to threatening stimuli. Interestingly, the effects of CM on amygdala responses may vary according to the type and timing of CM [29]: Amygdala responses to threatening stimuli were attenuated in adults reporting exposure to physical abuse between 3 and 6 years of age, but exacerbated in adults reporting exposure to emotional bullying at 13–15 years. The authors offer plausible explanations for these differential neuroplastic adaptations. Whereas a decreased fight-flight response may be adaptive for a child that is dependent on periodically physically abusive parents; for a teenager an increased fight-flight response may be more beneficial [30].

Recently, several scholars have returned to Karpman’s concept of secondary psychopathy [31] or acquired callousness [32], focusing on the experience of childhood psychological trauma. Earlier, Porter [33] had already provided an alternate causal pathway to the predominant view of a genetic predisposition to psychopathy of that time. He hypothesized that (secondary) psychopathy is the result of “‘de-activation’ or dissociation of a developing basic affective nature and conscience” (p. 183). This de-activation is viewed as a coping mechanism in response to traumatic interpersonal experiences. Porter [33] linked the affective blunting in psychopathy to the emotional numbing and feelings of detachment that are key diagnostic symptoms of Posttraumatic Stress Disorder (PTSD; [34]).

Other theoretical formulations, notably complex trauma [35] and Betrayal Trauma Theory (BTT; [36]) are also relevant in relation to the etiology of psychopathy. Complex trauma is defined as exposure to traumatic stressors at an age (e.g., early childhood) or in a context (e.g., prolonged torture or captivity) that compromises secure attachment with primary caregivers [37]. Complex trauma causes the organism to enter into “survival mode”, resulting in changes to many developing self-regulatory mechanisms [35], including: (a) attention and learning; (b) sensorimotor functions; (c) working (short-term processing), declarative (verbal) and narrative (autobiographical) memory; and (d) emotion regulation and social relatedness (attachment). BTT emphasizes the interpersonal context in which most early CM takes place, particularly intrafamilial abuse. Central to BTT is attachment theory, the notion that humans depend on others for physical survival and emotional responsiveness [38]. According to BTT, the degree to which the abuse represents a betrayal by the trusted and needed attachment figure mediates the way in which abuse-related information is processed and remembered [39]. Forgetting and misremembering of the abuse, as well as emotional dissociation, are hypothesized to be specifically linked to betrayal vs. other, nonbetrayal trauma [40].

In summary, there appears to be an increased interest in theoretical models that assign an etiological role to early CM in the development of psychopathy. Several theorists proposed models in which early maltreatment experiences, particularly those with primary caregivers, produce a blunted or dissociative response to stress, as a key factor in the affective deficits observed in psychopathy (e.g., [31, 32, 41]), whereas previously, the so-called ‘affective core’ of psychopathy was viewed as largely innate [23]. CM has also been linked to the type of externalizing symptoms represented by Factor 2 of psychopathy (e.g., [42]). If it can be demonstrated that environmental factors, such as CM, play a role in the development of psychopathy, these factors could be targeted for preventive efforts and treatment interventions.

### Empirical studies about the role of childhood maltreatment in psychopathy

Research on the relationship between CM and psychopathology, including psychopathy, is mired by a number of methodological difficulties. Ideally, CM would be measured prospectively and objectively, before the onset of psychopathy. However, in studies of adults, CM is mostly measured through retrospective self-report, which can be biased in the form of memory errors of both omission and commission [43–45]. Other research designs use official Child Protection Services records, but these are likely to underestimate the true prevalence of maltreatment [43]. As best-evidence practice, CM would be established through triangulation, using official records, collateral informants, and self-report, but this type of rigor is still quite rare.

The first studies that explored the hypothesized link between CM and psychopathy were case studies conducted in the 1940s (e.g., [16, 46]). Weiler and Widom [47] performed one of the first prospective studies that used the PCL-R in a large sample of young adults (*n* = 652) and compared them with a control group, matched for demographics and criminal history (*n* = 489). The abused and neglected group was composed of substantiated cases of childhood physical and sexual abuse and/or neglect processed in the county juvenile or adult criminal court and the subjects were 11 years or younger at the time of receiving maltreatment. Abused/neglected individuals had statistically significantly higher PCL-R total scores than matched controls, regardless of gender or ethnicity. Lang et al. [48] attempted to replicate the Weiler and Widom study with a Swedish sample, comprised of 192 boys aged 11–14 years accused of property crimes and 95 boys, matched for age, family type (separated or not), social group and neighborhood. Child abuse and neglect was determined at age 11–14 by means of triangulation, using school and social work records, parent and child interviews and self-report. The sample was followed up at age 32–40. Mean PCL-R scores of the high victimization group were statistically significantly higher than those of the low victimization group. Notably, neither of these studies examined associations between the four PCL-R facets and CM.

Another early study into the association between CM and PCL psychopathy used a purely retrospective design in a Scottish prison sample [49]. Fifty psychopaths (Mean PCL-R = 29) and 55 nonpsychopaths (Mean PCL-R = 13) were administered the Childhood Experience of Care and Abuse interview (CECA; [50]). The CECA asks about specific experiences and events occurring in childhood as opposed to subjective feelings. The authors found statistically significantly higher scores in the psychopathic group compared to the nonpsychopathic group on parental discipline, parental antipathy, parental neglect, parental control, and psychological abuse. No differences were found for physical and sexual abuse. Using multiple regression analysis, Marshall and Cooke [49] found that victimization within the family statistically significantly predicted PCL-R Factor 1 scores, whereas societal adversity (school experience, school performance, institutional stay) was the main predictor of PCL-R Factor 2 scores. This was one of the first studies that showed a specific effect for childhood family trauma on the Affective-Interpersonal factor. However, a study that used a relatively similar design (a sample of 615 male American offenders, retrospective reports of child abuse/neglect and PCL-R rated psychopathy) did not replicate this finding [51]. Using the Cooke and Michie [10] 13-item model, these authors found a statistically significant association of CM with the behavioral lifestyle factor, but not with the affective and the interpersonal factors [51].

#### Gender differences

Psychopathy manifests itself differently in women compared to men [52]. High psychopathy scores in women correlate with more emotional dysregulation as well as manipulative and sexualized behaviors [53, 54]. Fewer studies have explored the link between CM and psychopathy in female subjects, as compared to male subjects. Overall, statistically significant associations between different forms of CM and psychopathy have been shown in females (for an overview, see [52]). However, while some studies found stronger associations between CM and psychopathy for women, in other studies such associations were stronger for men [55, 56]. As findings are currently inconclusive, it remains an open question whether CM is similarly related to psychopathic traits in men and women.

### The present study

Given the current theoretical models and prior empirical research on the link between CM and psychopathy, we deemed it appropriate to conduct a meta-analysis of this literature. We chose to include both Hare PCL-derived scales and self-report instruments, which have been used most prominently in the field. We believed this would on the one hand cast the widest net in terms of allowing us to include a large number of studies, and on the other hand give us the possibility to gain insight into the link between the four facets of psychopathy and CM. We opted to include all types of CM, but did not include other indicators of childhood adversity, such as socioeconomic adversity or community violence, although we acknowledge that these may act as risk factors for antisocial and psychopathic behavior [57].

By conducting a meta-analysis of the existing research base, we aimed to find answers to the following questions:

1. Is there a statistically significant association between any type of CM and psychopathy in general and as defined by its two factors and four facets?
2. Do different types of CM (neglect vs. physical vs. sexual vs. emotional abuse) have similar associations with psychopathy and its factors and facets?

We were also interested in conducting several moderator analyses:

3. Does gender (male vs. female) impact the link between psychopathy and CM?
4. Does the psychopathy measure used (PCL-scale vs. other psychopathy measure) impact the link between psychopathy and CM?
5. Does the type of publication (peer reviewed journal articles vs. grey literature) impact the link between psychopathy and CM?
6. Does study design (prospective vs. retrospective) impact the link between psychopathy and CM?
7. Does sample type (clinical/correctional vs. community) impact the link between psychopathy and CM?

Because there is currently no comprehensive theoretical framework on the relationships between specific types of CM and the different psychopathic traits, our meta-analysis is mostly exploratory. However, we did formulate a few specific hypotheses, which we believed could be supported by the current literature.

H1: Factor 1 is related to CM in general, and emotional abuse/neglect in specific, based on the ‘deactivation’ hypothesis (e.g., [33]) and prior research (e.g., [58]) showing that emotional abuse by attachment figures in childhood is likely mediated by neurocognitive adaptations that result in ‘blunted’ affective responsiveness to threat [33, 58]. Thus, we predict that childhood emotional abuse will relate most strongly to Factor 1 (including both the affective and the interpersonal facets).

H2: Factor 2 is related to CM in general, and physical abuse in specific, based on a large body of prior research (e.g., [42, 59]) that demonstrated that anger, impulsivity, and antisocial acting out are related to childhood physical abuse by attachment figures, likely mediated by neurocognitive changes that result in increased stress reactivity. We predict that childhood physical abuse will relate most strongly to Factor 2 (including both the lifestyle and the antisocial facets).

## Method

### Protocol and open data

This meta-analysis was pre-registered on March 26, 2018, under the PROSPERO platform, an international prospective register of systematic reviews. The registration can be found on their webpage (https://www.crd.york.ac.uk/PROSPERO/) with the ID: CRD42018091678. Furthermore, the raw data are accessible under the Open Science Framework (OSF; https://osf.io/2kqcw/).

### Inclusion criteria

Our variables of interest were psychopathy, measured by means of self-report or clinical judgment, and CM as defined by the World Health Organization (WHO):

Child maltreatment is the abuse and neglect that occurs to children under 18 years of age. It includes all types of physical and/or emotional ill-treatment, sexual abuse, neglect, negligence and commercial or other exploitation, which results in actual or potential harm to the child’s health, survival, development or dignity in the context of a relationship of responsibility, trust or power. Exposure to intimate partner violence is also sometimes included as a form of child maltreatment [60].

The following criteria were used to include studies: (1) peer-reviewed articles, grey literature, book chapters/books and dissertations; (2) prospective and retrospective studies; (3) studies reported in the English language. Studies needed to include: (1) community, clinical, or correctional samples of children or adults (male and female) in which both CM and psychopathy were measured; (2) CM: physical abuse, emotional abuse, sexual abuse, and neglect, measured by means of self-report and/or official reports; (3) psychopathy measured by means of self-report or clinician rated scales.

### Exclusion criteria

We excluded: (1) single-case studies, conference abstracts without primary study data, book reviews; (2) papers not written in English; (3) studies which focused on childhood adversities outside the family or primary caretaking environment, such as war trauma or community violence; (4) studies on other types of childhood adversity, such as loss of a parent or serious accidental injury, or traumatic experiences after age 18; and (5) studies without an objective measure of psychopathy.

Unlike initially specified in our PROSPERO registration, studies that focused solely on callous-unemotional (CU) traits were excluded from the current review. This decision was made as the number of located articles investigating psychopathy exceeded our expectation so that the inclusion of CU traits would only add heterogeneity to the synthesis of effect sizes.

### Literature search

Consistent with the Preferred Reporting Items for Systematic Review and Meta-Analysis guidelines (PRISMA; [61]), a systematic literature search of papers published between January 1990 and January 2021 was conducted. The year 1990 was chosen because the first validation study of Hare’s revised version of the Psychopathy Checklist (PCL-R; [62]) was published in 1990. The first search was conducted in February 2018 on PsycINFO, PubMed, and Web of Science and subsequently updated in January 2021. The following keywords were used: (trauma* OR complex trauma* OR childhood trauma* OR abus* OR *adverse childhood experience*+ OR *neglect* OR *maltreatment* OR *betrayal trauma*) AND (*psychopath*+ OR *psychopathic* OR *psychopathy* OR *callous unemotional trait*+ OR *CU trait*+ OR *CU-trait*+ OR *ODD* OR *CD* OR *conduct disorder*+ OR *oppositional defiant disorder*+ OR PCL* OR *antisocial* OR *dissocial*).

To identify all potential and ongoing research, the project was uploaded to ResearchGate (https://www.researchgate.net/project/The-link-between-childhood-trauma-and-psychopathy-A-systematic-review). Additionally, researchers who had previously conducted research on psychopathy were contacted via email. Covidence software (www.covidence.org) was utilized to aid the process of the systematic review management and collaboration.

### Study selection

The screening process was conducted in two stages. At each stage, two members of the research team independently assessed whether a publication should be included or excluded for further analysis. A consensus was reached if both assessors agreed on the inclusion or exclusion of the publication. Conflicts were resolved by the first author (CdR). Initially, studies were screened for eligibility by examining their title and abstract. Subsequently, eligible studies were assessed via full text screening. A number of dissertations and grey literature that could not be accessed from open sources were retrieved by contacting the University library in which the dissertation was defended or by contacting the authors through LinkedIn or other social media.

### Data collection

The following information was retrieved from all studies that met the aforementioned inclusion criteria: type of publication; sample type; sample mean age and standard deviation; sample sex distribution; country of study; total sample size; CM measure; and psychopathy measure. On occasion, not all of the above data could be obtained from the text. In such instances, the corresponding author of the paper was contacted for necessary information. Data extraction per study was always carried out by a pair of authors who had to be in agreement. Effect size extraction was conducted by the second author (MB).

### Summary measure

The Pearson product-moment correlation coefficient *r* was chosen as the effect size index because most studies reported correlational data to quantify the association between CM and psychopathy. If possible, coefficients were extracted directly from zero-order correlation matrices. Missing effect sizes were acquired either by contacting the corresponding authors or by estimating their magnitude from statistical information provided in the paper, such as *t*-statistics or *d* values [63]. For three studies [64–66], correlation coefficients had to be imputed from standardized regression coefficients using the equation *r* = .98*β* + .05*λ*, where *λ* equals 0 when *β* is negative and 1 when *β* is positive [67].

Given that each paper could report more than one effect size, the following approach was used to ensure independence among effect sizes [68]: (1) if a study reported data on two distinct samples (e.g., for men and women), the effect sizes were considered independent rather than combined into a composite effect size; (2) in cases where an author published multiple manuscripts, but used samples that were clearly drawn from the same population, only the study with the largest sample was included; (3) if applicable, separate effect sizes for general maltreatment, physical abuse, emotional abuse, sexual abuse, and physical and emotional neglect were derived from the same paper; (4) similarly, effect sizes for psychopathy total scores as well as for psychopathy factors and facets were extracted; and (5) composite effect sizes for psychopathy were calculated following the recommendations of Borenstein et al. [68] when multiple psychopathy measures were used or when estimates were only reported for psychopathy factors.

In an attempt to address the attenuation of correlation coefficients caused by measurement error [69], additional corrected effect sizes and variances were computed. The required reliability estimates were retrieved from the included articles, or if the information was lacking, they were imputed using the *Mice* package in R by averaging the results of five imputed data sets [70].

### Meta-analysis

Because the distribution of correlation coefficients tends to be skewed in studies with a small sample size [71], the raw effect sizes were first transformed into Fisher’s *z*. All analyses were carried out using the transformed values, but were subsequently transformed back to correlation coefficients for presentation of the results. In addition, as studies were expected to vary fundamentally, random-effects models were chosen to calculate the average summary effect sizes [68]. The between-study variance *τ*^2^ was estimated with restricted maximum likelihood method (REML) and used to assign weights to each study by the inverse of total variance: $w_{i}=\frac{1}{\left(v_{i}+\tau^{2}\right)}$. Simulations have shown that REML tends to be less biased than other popular methods [72]. While *τ*^2^ reflects the variance of true effects in absolute terms, *I*^2^ was examined to quantify the relative amount of true heterogeneity among the total variability across studies. By convention, *I*^2^ values of 25%, 50%, and 75% respectively indicate low, moderate, and high levels of inconsistency in a meta-analysis [73].

Five separate meta-analyses were performed, for general maltreatment, physical abuse, emotional abuse, sexual abuse, and neglect. Moreover, a priori specified moderator analyses were conducted to determine whether the association between CM and psychopathy differed by (1) proportion of women in the sample; (2) type of sample (clinical/correctional vs. community sample); (3) psychopathy measure (PCL-scale vs. other psychopathy measure); and (4) type of publication (journal article vs. grey literature). Mixed-effect meta-regression models were fitted with categorical moderators included as dummy variables and the proportion of women as a continuous variable (ranging from 0 to 100%).

Given the multifaceted structure of psychopathy [74], additional subgroup analyses were carried out to examine whether the summary effect sizes vary across psychopathy factors and facets (i.e., affective, interpersonal, lifestyle, and antisocial). That is, each factor/facet was included as a subgroup and tested for between-group differences (H_0_: *r*_factor1_ *= r*_factor2_ and H_0_: *r*_affective_ *= r*_interpersonal_ *= r*_lifestyle_ *= r*_antisocial_; [75]). Statistically significant differences between psychopathy facets were then further investigated with post-hoc analyses contrasting *r*_affective_ vs. *r*_lifestyle_; *r*_affective_ *vs*. *r*_antisocial_; *r*_interpersonal_ vs. *r*_lifestyle_; *r*_interpersonal_ vs. *r*_antisocial_. Because most studies reported effect sizes for the two factors and/or four facets, three-level random effects models were used. Multi-level meta-analyses allow for dependencies among effect sizes by breaking *τ*^2^ down into the variance within samples (*σ*^2^_level2_) and the variance between samples (*σ*^2^_level3_; [76]).

To test the robustness of the results, included studies were assessed for influential effect sizes by the inspection of multiple influence measures (DIFFITS, Cook’s distance, covariance ratio) generated by the leave-one-out method [77]. Subsequent sensitivity analyses were performed to determine whether the deletion of such influential cases would alter the overall findings of this meta-analysis. If not, the results can be considered robust [77]. All analyses were performed with the *metafor* package [78] in the latest version of R 4.0.3 [79].

### Publication bias

As with any meta-analysis, publication bias poses a risk to the interpretability of the pooled effect size [80]. Therefore, its presence and impact were examined in three ways. First, funnel plots were created and inspected visually for asymmetry. An asymmetrical distribution of studies across the mean effect size may suggest publication bias [81]. Second, Egger’s linear regression method was used to test for a linear relationship between the effect size and its standard error, which provides a less subjective measure of asymmetry in a funnel plot [82]. Third, Duval and Tweedie’s Trim and Fill method was applied to estimate an unbiased summary effect size by imputing missing effect sizes [83]. This approach allows one to quantify the impact of reporting bias on the observed result [84].

## Results

### Descriptive results

**Fig 1:**
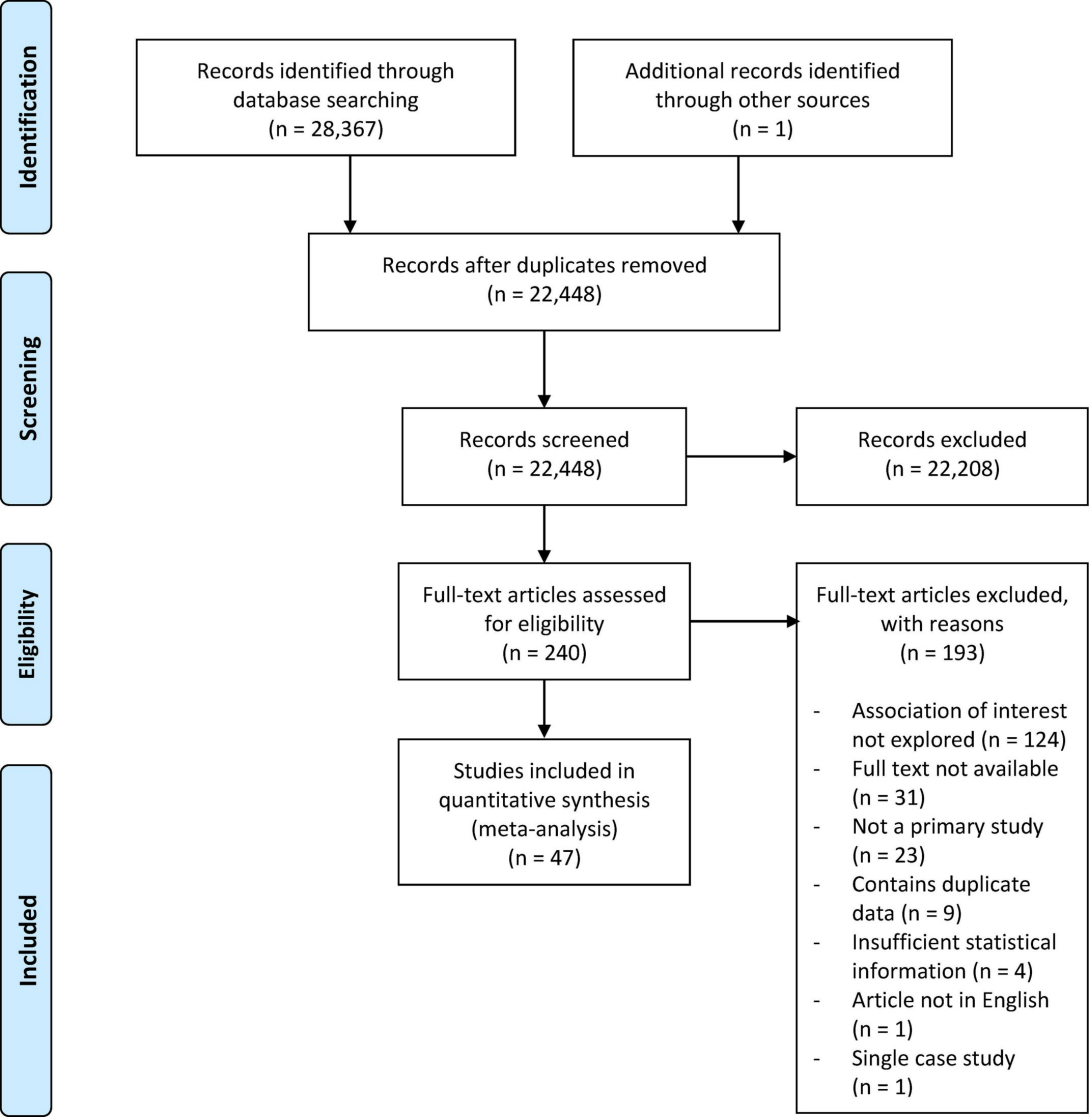
PRISMA flowchart showing the study selection process.

Fig 1 provides a visual outline of the study selection process. Our literature search generated 28,368 papers. Subsequently, 5,920 duplicates were removed. The remaining 22,448 papers entered the title and abstract screening stage, in which 22,208 were excluded in accordance with the inclusion and exclusion criteria specified in the Method section. A total of 240 papers entered the full text screening stage, in which 193 were excluded. The reasons for exclusion were the following: The association between primary variables of interest was not explored (*n* = 124), full text not available (*n* = 31), not a primary study (*n* = 23), contains duplicate data (*n* = 9), insufficient statistical information to calculate effect size (*n* = 4), full text not in English (*n* = 1), and single case study (*n* = 1). The final sample included in the meta-analysis comprised 55 independent samples and a total of 389 effect sizes originating from 47 papers.

#### Study and sample characteristics

All papers were published between 1996 and 2020. Forty-three of the included papers were journal articles and four were dissertations. The included papers originated from six continents, namely North America (*n* = 32), Europe (*n* = 11), South America (*n* = 1), Africa (*n* = 1), Asia (*n* = 1), and Australia (*n* = 1).

A total of 12,737 participants were included in the final set of articles, where sample sizes ranged from relatively small (*n* = 22; [85]) to very large (*n* = 1,169; [86]). The included papers predominantly used correctional or clinical samples (*n* = 34), though some papers used samples from the general population (*n* = 13). Twenty-one study samples were solely male, five were female, and 21 were mixed. Further details on study and sample characteristics are given in S1 Table.

#### Measure characteristics

The included papers used a range of measures to explore the variables of interest, namely CM and psychopathy. To assess CM, most papers used retrospective self-report measures, such as the Childhood Trauma Questionnaire (CTQ; [87]). A minority of papers used official child welfare records as a measure of CM. Few papers used a combination of self-report measures and official records. For the assessment of psychopathy, most papers employed a clinician-rated scale from the family of Hare Psychopathy Checklist measures, such as the PCL-R [13, 23], PCL-SV [88], or PCL:YV [89]. A minority of papers used self-report psychopathy scales, such as the Self-Report Psychopathy Scale (e.g., SRP; [90]) or the Youth Psychopathy Traits Inventory (YPI; [91]). None of the included papers used a combination of clinician-rated and self-report measures to assess psychopathy.

### Meta-analytical results

Statistically significant positive associations were found between all five types of CM and the total psychopathy score. The pooled effect size for sexual abuse was smaller than for the other four types of maltreatment, but still statistically significant. Heterogeneity between studies was moderate to high. After excluding influential studies, effect sizes increased slightly and heterogeneity decreased considerably. The results are presented in Table 1. Corresponding figures, such as forest plots (S1–S5 Figs) and funnel plots (S6 Fig) can be found in our supporting material. In addition, we tested a multi-level model in which all effect sizes were included simultaneously (instead of five separate meta-analyses). Because this model yielded similar results, these are reported only in the S2 Table.

**Table 1:** Meta-analytical results of the association between childhood maltreatment and psychopathy.

Type of maltreatment	*k*	*r*	SE_*r*_	95% CI	*Q*-test	*I*^2^ (%)
General maltreatment	32	.20***	.02	[.16, .24]	65.69***	57.36
	(31)	(.21)***	(.02)	([.18, .24])	(51.70)**	(42.31)
Physical abuse	32	.19***	.02	[.16, .22]	46.62*	36.82
	(31)	(.20)***	(.01)	([.17, .22])	(30.22)	(.52)
Emotional abuse	25	.15***	.03	[.09, .20]	87.76***	71.08
	(24)	(.17)***	(.02)	([.13, .21])	(40.80)*	(46.00)
Sexual abuse	32	.10***	.02	[.06, .14]	70.06***	57.55
Neglect	20	.21***	.02	[.17, .26]	51.24***	58.57

Results without influential cases are in parentheses. *k* = number of effect sizes; *r* = pooled correlation; SE_*r*_ = standard error of *r*; CI = confidence interval of *r*; *I*^2^ = proportion of true heterogeneity; *Q*-test = *Q*-test for heterogeneity.

***p < .001.

**p < .01.

*p < .05.

### Correction for attenuation

Fitting the meta-analytical models with correlation coefficients corrected for attenuation produced only negligibly larger pooled effect sizes, with *r*_c_ = .24 (95% CI [.19, .28]), *r*_c_ = .22 (95% CI [.18, .26]), *r*_c_ = .17 (95% CI [.11, .23]), *r*_c_ = .11 (95% CI [.07, .15]), and *r*_c_ = .26 (95% CI [.19, .33]) for general maltreatment, physical abuse, emotional abuse, sexual abuse, and neglect, respectively.

### Moderator analyses

The proportion of women in a sample, the type of psychopathy measure used (i.e., PCL-scale vs. other), and the type of publication (i.e., peer-reviewed journal article vs. grey literature) did not statistically significantly moderate the association between CM and psychopathy. The type of sample (i.e., clinical/correctional vs. community), on the other hand, revealed evidence for moderation (*Q*(1) = 6.50, *p* = .0108). Clinical and correctional samples showed smaller, but still statistically significant, positive associations between general maltreatment and psychopathy (Clinical/Correctional: *r* = .17 [.12, .21]) compared to community samples (Community: *r* = .26 [.20, .31]). The moderator analysis for prospective vs. retrospective studies of CM could not be carried out due to a lack of prospective studies.

### Analyses for the four facets and two factors of psychopathy

Analyses on the facets and factors of psychopathy were conducted on a limited set of studies (facets: *k* between 9 and 11; factors: *k* between 2 and 7). For the psychopathy facets, the three-level random effects models revealed different patterns for the different types of CM (Table 2). General maltreatment, physical abuse, and neglect showed statistically significant associations with all four facets, although of different magnitudes. Emotional abuse and sexual abuse were statistically significantly correlated with some, but not with all of the four psychopathy facets. Statistically significant between-group differences across facets were found for general maltreatment, physical abuse, and emotional abuse. Subsequent post-hoc analyses indicated a stronger relationship between CM and the lifestyle and antisocial facets compared to the affective and interpersonal facets. These findings are presented in Table 2.

**Table 2:** Between-group differences among psychopathy facets by each type of childhood maltreatment.

							Post-hoc comparisons (*z*-value)			
	*k*	*r*	SE_*r*_	95% CI	*Q*_between_	*Q*_within_	aff vs. lif	aff vs. ant	int vs. lif	int vs. ant
General maltreatment					25.12***	74.53***	3.00**	3.23**	3.76***	4.00***
--Affective	11	.11***	.03	[.05, .17]						
--Interpersonal	11	.08*	.03	[.02, .15]						
--Lifestyle	11	.21***	.03	[.15, .28]						
--Antisocial	11	.22***	.03	[.16, .28]						
Physical abuse					16.00**	37.06	1.42	3.78***	0.66	3.02**
--Affective	11	.09**	.03	[.03, .15]						
--Interpersonal	11	.12***	.03	[.06, .18]						
--Lifestyle	11	.14***	.03	[.08, .20]						
--Antisocial	11	.23***	.03	[.17, .28]						
Emotional abuse					12.33**	58.10*	2.58**	1.86	3.13**	2.40*
--Affective	10	.07	.04	[.01, .15]						
--Interpersonal	10	.05	.04	[–.03, .13]						
--Lifestyle	10	.17***	.04	[.10, .25]						
--Antisocial	10	.15***	.04	[.07, .22]						
Sexual abuse					06.14	39.24	–	–	–	–
--Affective	11	.00	.03	[–.06, .07]						
--Interpersonal	11	.03	.03	[–.03, .09]						
--Lifestyle	11	.09**	.03	[.03, .15]						
--Antisocial	11	.03	.03	[–.03, .09]						
Neglect					03.47	26.89	–	–	–	–
--Affective	9	.13***	.03	[.06, .19]						
--Interpersonal	9	.12***	.03	[.06, .18]						
--Lifestyle	9	.18***	.03	[.11, .23]						
--Antisocial	9	.18***	.03	[.12, .24]						

*k* = number of effect sizes; *r* = pooled correlation; SE_*r*_ = standard error of *r*; CI = confidence interval of *r*; *Q*_between_ = variance accounted for by subgroups; *Q*_within_ = residual heterogeneity in effect size; aff = affective; int = interpersonal; lif = lifestyle; ant = antisocial.

***p < .001.

**p < .01.

*p < .05.

Similar results were found for the two psychopathy factors. All types of CM showed larger effect sizes for Factor 2 than for Factor 1. However, given the low power due to the limited number of studies, differences between the two factors were statistically significant only for general maltreatment and sexual abuse (Table 3).

**Table 3:** Between-group differences among psychopathy factors by each type of childhood maltreatment.

	*k*	*r*	SE_*r*_	95% CI	*Q*_between_	*Q*_within_
General maltreatment					6.46*	11.51
Factor 1	7	.15***	.03	[.09, .21]		
Factor 2	7	.26***	.03	[.20, .31]		
Physical abuse					2.93	2.61
Factor 1	4	.19***	.04	[.11, .26]		
Factor 2	4	.27***	.04	[.20, .34]		
Emotional abuse					.99	8.24
Factor 1	3	.13	.08	[–.03, .29]		
Factor 2	3	.19*	.08	[.03, .35]		
Sexual abuse					6.24*	8.16
Factor 1	4	.07	.05	[–.03, .16]		
Factor 2	4	.20***	.05	[.10, .29]		
Neglect					.65	3.89
Factor 1	2	.19	.11	[–.02, .39]		
Factor 2	2	.26*	.11	[.06, .45]		

*k* = number of effect sizes; *r* = pooled correlation; SE_*r*_ = standard error of *r*; CI = confidence interval of *r*; *Q*_between_ = variance accounted for by subgroups; *Q*_within_ = residual heterogeneity in effect size.

***p < .001.

**p < .01.

*p < .05.

### Publication bias

The visual inspection of funnel plots provided no indication of asymmetry (S6 Fig). This was corroborated by statistically nonsignificant Egger’s regression tests for all types of CM. Furthermore, Duval and Tweedie’s Trim and Fill method imputed missing studies for physical abuse (*k* = 1), sexual abuse (*k* = 6), and neglect (*k* = 2) (S6 Fig), resulting in slightly larger unbiased pooled effect sizes: *r*_unbiased_ = .19 (95% CI [.15, .22]), *r*_unbiased_ = .12 (95% CI [.08, .17]), and *r*_unbiased_ = .23 (95% CI [.17, .28]), respectively. Overall, these results speak against the presence of publication bias.

## Discussion

This meta-analysis was conducted against the background of past and present-day theories about the role of CM in the possible etiology of psychopathy. Theorists have offered diverging views on the purported link between CM and the different components of psychopathy. Some (e.g., [16, 92]) have proposed a link between CM and the behavioral disinhibition/impulsivity aspect of psychopathy, whereas others (e.g., [31, 33]) also hypothesized an association between the affective component and CM. As such, the separate components of psychopathy are viewed as the result of distinct symptomatic responses to psychological trauma, with psychopathic behavioral disinhibition linked to increased arousal and reactivity (cf. DSM-5 criterion E for PTSD; [34]), and psychopathic lack of affect linked to emotional numbing/avoidance/dissociation (DSM-5 criterion C for PTSD; [34]).

There is currently no overarching theory of the associations between specific types of CM and the psychopathy factors and facets. Thus, our meta-analysis is largely exploratory, although we also tested two specific hypotheses. First, the association between general CM and psychopathy was *r* = .20 (*r*_c_ = .24). For the specific types of CM, the associations were *r* = .19 (*r*_c_ = .22) for physical abuse, *r* = .15 (*r*_c_ = .17) for emotional abuse, *r* = .10 (*r*_c_ = .11) for sexual abuse, and *r* = .21 (*r*_c_ = .26) for neglect. There has been discussion in the field on how to interpret effect size *r*. Cohen [93] recommended Pearson *r* values of .10, .30, and .50 to demarcate small, medium, and large effects, respectively. However, recent research has cast doubt on this classification. A review of studies by Gignac and Szodorai [94], based on 708 meta-analytically derived correlations, reported that the 25th, 50th, and 75th percentiles corresponded to correlations of .11, .19, and .29, respectively. Fewer than 3% of correlations met Cohen’s definition of ‘large’ (i.e., .50 or higher). Gignac and Szodorai [94] suggest that in real life, the terms small, medium and large more closely correspond to correlations of .10, .20 and .30. If we take the latter values as benchmarks, psychopathy has a medium sized association with physical abuse, neglect, and CM in general, and a small association with childhood emotional abuse and sexual abuse. This is in line with Götz et al. [95], who propose that for complex psychological constructs (like psychopathy) small effects are the norm rather than the exception given that these constructs are typically caused and influenced by a multitude of factors.

Second, our first, ‘emotional numbing’ hypothesis assumed an association between Factor 1 (and its two facets) with emotional abuse and neglect in specific. This hypothesis was only partially supported (see Tables 2 and 3). We did not find statistically significant associations between emotional abuse and Factor 1 and its interpersonal and affective facets. However, neglect was statistically significantly related to the interpersonal and affective facets (but not the overall Factor 1, which is likely due to the limited number of studies). It should also be noted that Factor 2 (and its two facets) did show a statistically significant, and even larger association with neglect compared to Factor 1 (*r* = .19 for Factor 1 versus *r* = .26 for Factor 2).

Child neglect is defined as a failure of the child’s caregivers to meet its needs with regard to food, clothing, shelter, supervision, education, affection, or medical care [96]. Child neglect is extremely broadly defined and that may be one of the reasons it is the most prevalent form of CM [97, 98], with effects on academic and social-emotional development [99]. The exact dynamics and mechanisms whereby childhood neglect leads to short- and long-term consequences, including the affective and interpersonal facets of psychopathy, have not been well-studied [100]. A meta-analysis of 30 observational studies of abused, neglected, and nonmaltreated children’s behavior during interactions with their parents provides some insights into possible underlying dynamics [101]. Findings showed that neglected children had statistically significantly lower ‘involvement’ (defined as attention, interest) compared to nonmaltreated children (moderate mean weighted effect size *d* = .75), whereas the difference in level of involvement between abused and nonmaltreated children was only small (*d* = .39). Interestingly, the abused and neglected children did not differ meaningfully on the behavioral dimensions of aversiveness (anger, resistance) and positivity (affection, approval): Both neglected and abused children showed similar effects in comparison to nonmaltreated children (*d*’s ranging between .29 and .51, all in a less favorable direction for the abused/neglected children; [101]). This meta-analysis shows that parental neglect results in increased emotional noninvolvement in children, which is in line with the affective blunting hypothesis. A study into the effects of childhood abuse and neglect on adult cognitive functioning provides additional evidence in support of this idea [102]. The authors found a statistically significant association between emotional processing inhibition, as measured with the Cambridge Neuropsychological Test Automated Battery (CANTAB; [103]) and neglect, but not with abuse. Still, all of these studies are correlational, and do not provide conclusive evidence.

Third, our second hypothesis concerned the relationship between physical abuse and Factor 2 and its lifestyle and antisocial facets. This hypothesis is clearly supported by our meta-analytic results: Factor 2 shows the strongest correlation with physical abuse (*r* = .27) of all types of CM (see Table 3). Both the lifestyle and the antisocial facets show statistically significant associations with physical abuse, the latter being the higher (*r* = .14 and .23, respectively). Numerous prior studies have shown a relationship between physical child abuse and externalizing behavior problems, including antisocial/criminal behavior (e.g., [104–107]). Many developmental psychological theories have suggested mechanisms by which physical abuse by caregivers increases the child’s risk to develop problems with emotion regulation, impulse control, and ultimately also antisocial and criminal behavior, often mediated by deviant peer socialization: attachment theory (e.g., [108–110]), social cognition theory (e.g., [111, 112]) and social interaction learning theory (e.g., [113]). As mentioned in the Introduction, the emotional and behavioral dysregulation characteristic of Factor 2 has been empirically linked to neurocognitive abnormalities in relevant brain regions, such as the dorsolateral prefrontal cortex and the medial orbitofrontal cortex [114].

Finally, it should be noted that the associations for the lifestyle and antisocial facets (and Factor 2) were stronger than for the affective and interpersonal facets (and Factor 1), with the exception of neglect and sexual abuse. Of note, sexual abuse only showed a statistically significant but small correlation with the lifestyle facet. The summary effect sizes of general CM, physical abuse, and neglect were generally larger, and mostly in the moderate range for the lifestyle and antisocial facet, and in the small range for the affective and interpersonal facet. It should be noted that the facet analyses were conducted on a much smaller sample of effect sizes, which limits the robustness of these specific findings. Definitive conclusions regarding the link between different types of CM and the four facets and the two factors of psychopathy should be deferred until further studies become available.

Our moderator analysis showed that gender of study participants, type of psychopathy measure (PCL-scale vs. other) used and type of publication (peer-reviewed vs. grey literature) did not impact the association between psychopathy and CM. As such, a reasonable inference is that the CM-psychopathy relationship is relatively robust across levels of these moderators, including gender, psychopathy measure, and publication type. We should mention that the number of effect sizes per subgroup (*k*) for some of these moderator analyses was rather small, which limits the robustness and interpretability of our findings. We found statistically significant moderation for sample type: correctional and clinical samples showed smaller, but still statistically significant, correlations between psychopathy and general CM compared to general community samples (.17 vs. .26). The interpretation of this finding is not straightforward. On one hand, one could argue that the opposite effect would have been more logical, because psychopathic traits and CM are less prevalent in community samples than in clinical and correctional samples. On the other hand, the spread (i.e., variance) of both CM and psychopathic traits may be larger in the community than in clinical/correctional samples, leading to stronger correlations. Interestingly, our results appear in line with the meta-analytic findings of Douglas et al. [115] who studied the association between psychotic symptoms and violence. They found that this relationship was statistically significantly stronger in community samples compared to psychiatric and correctional samples.

Our meta-analysis points at significant gaps in the research literature: most studies have been conducted in institutionalized clinical/correctional samples, using retrospective self-report of CM. Therefore, it is possible that memory distortion (so-called recall bias) may impact the strength of the association between psychopathy and CM. Prospective studies continue to be rare, likely because they require extreme efforts and are thus, costly. A relevant example is a longitudinal study by Shi et al. [116], which found that actual, observed maternal withdrawal in response to an 18-month old infant’s distress (cf., emotional neglect), statistically significantly predicted features of Antisocial Personality Disorder (ASPD) 20 years later. Although not identical, ASPD and psychopathy are similar, and it is interesting to note how a study with a longitudinal design that used such a long follow-up period provides support in line with our hypothesis.

An important issue in meta-analytic research is whether the reported findings are robust and valid. The number of studies included in the present meta-analysis is reasonably high (between 20 and 32). We also succeeded in including studies from different continents, although North-American studies dominated. Both the number of included studies and their global representativeness increase our trust in the robustness of the effects. In addition, the robustness of our findings was further supported by our sensitivity analysis suggesting that our results are not substantially impacted by a few influential cases. A common threat to the validity of meta-analytic findings is the file-drawer problem. Published research studies may overestimate the true effect sizes if journals prefer to accept papers that report strong statistically significant associations over papers with statistically nonsignificant or small effects [63]. Comparisons of the results of published and unpublished studies as well as the trim and fill procedure [83] and Egger’s regression test for funnel plot asymmetry [82] indicated that the file drawer issue was not a major concern in our meta-analysis.

### Implications for theory and clinical practice

The present meta-analysis provides support for a moderate link between psychopathy and childhood physical abuse, emotional abuse, and neglect, as well as overall CM. The link between psychopathy and childhood sexual abuse is small. These associations are stronger for the lifestyle and antisocial facets than for the affective and interpersonal facets of psychopathy, but nearly all associations are statistically significant. Our findings are in line with theories of the impact of complex trauma [35] and BTT [36] on the development of serious personality pathology, although most previous theorizing and empirical research have focused on Borderline Personality Disorder (for a review, see [117]) and not psychopathy. An exception to this is a recent study by Yalch and Levendosky [118] who found that exposure to trauma high in betrayal was the only predictor of the vulnerable and grandiose dimensions of pathological narcissism in a college student sample, after controlling for other forms of (nonbetrayal) trauma exposure. The authors suggest “that not only does exposure to high betrayal trauma inflict a psychological wound (narcissistic vulnerability), but also that it influences the means by which people defend against that wound (narcissistic grandiosity)” [118]. Narcissistic grandiosity resembles the interpersonal facet of psychopathy.

During the past decade, researchers have become increasingly aware of the importance of CM as a risk factor for psychopathology generally (for recent reviews, see for example: [30, 119]). The notion that CM causes phenotypic alterations to the developing brain [120, 121], which are subsequently impacted by further epigenetic and developmental changes, is starting to impact psychiatric nosology [30]. In recent years, new diagnostic classifications, such as developmental trauma disorder [122] and complex PTSD [123, 124] have been developed. Teicher [30] is highly optimistic that revising the DSM to acknowledge the critical role of CM “would be a quantum step forward in the development of an etiologically informed classification system” (p. 5). The current psychiatric classification system focuses entirely on symptoms without regard to etiology, pathophysiology, or theory, which results in pathophysiologically heterogeneous disorders with similar clinical presentations being considered as unitary disorders. At the very least, the statistically significant association between CM and psychopathology should prompt mental health professionals to include trauma-informed assessment and treatment in their standards of (forensic) clinical practice [125, 126].

Recently, McCrory and colleagues [127] reviewed the existing functional neuroimaging research of children and adolescents exposed to maltreatment across four neurocognitive domains: threat processing, reward processing, emotion regulation, and executive control. They discussed these findings within the context of their “theory of latent vulnerability”, which conceptualizes the link between childhood maltreatment and risk of mental disorder across development [128]. According to this theory, maltreatment results in measurable alterations in several neurobiological systems that reflect adaptation to early abusive environments. In principle, these adaptations are often beneficial within the early adverse environment, but they are thought to incur long term disadvantages as they may mean that the individual is poorly equipped to adapt to more normative environments. Studies on processing of threatening stimuli show increased as well as decreased neural responsiveness in maltreated samples, particularly in the amygdala, thought to reflect hypervigilance and avoidance, respectively. Studies on reward processing report diminished neural responsiveness to anticipation and rewards, especially in the striatum. Research on emotion regulation reports increased activation of the anterior cingulate cortex (ACC) during active emotion regulation, possibly reflecting greater effortful processing. Finally, studies of executive control, although limited in number, report increased dorsal ACC activity during error monitoring and inhibition. All of these neurobiological systems have also been implicated in the development of psychopathic traits [129]. Future research into the neurobiological underpinnings of psychopathic traits should include assessment of childhood maltreatment, preferably using prospective and objective measures of maltreatment, such as records from child protection services, to avoid a premature conclusion on the ‘innateness’ of certain psychopathic traits [20].

The view that at least some of the signature features of psychopathy can be seen as responses to complex or betrayal trauma, that is, repeated incidents of maltreatment over an extended period of time (i.e., months or years) which includes emotional abuse, physical abuse, neglect, and/or witnessing family violence within the caregiver system, provides further direction for preventive and therapeutic efforts. Therapeutic interventions that focus on early childhood trauma can provide a test of the causal role of trauma, if it could be shown that processing of traumatic experiences leads to meaningful reductions in psychopathic traits.

Several trauma-focused treatment interventions for psychopathy have been developed: Dialectical Behavior Therapy for psychopathy [130] and Schema Therapy (ST) for forensic patients with personality disorders, including psychopathy [131]. Controlled effectiveness studies of these therapy models have not yet been published, but a single case study documented the process of individual ST in a Dutch forensic patient with psychopathic traits [132]. This patient had been a victim of extreme physical and emotional abuse as a child and the therapist used different ST techniques (e.g., limited reparenting, experiential techniques) to alter the patient’s maladaptive schema modes. The case study also showed the patient’s PCL-R total score changed from 27 at baseline to 14 after four years of intensive ST. Remarkably, the Affective facet showed the largest change: from 7 to 1; the Interpersonal facet decreased from 4 to 1. This finding, although just an *N* = 1 result, challenges the notion that affective and interpersonal features of psychopathy are immutable ([133]; see [134] for further argumentation).

### Limitations

The findings of this systematic and meta-analytic review should be considered in light of a number of limitations. First and foremost, correlational analyses, such as those conducted in this meta-analysis, cannot be used to prove causality. Shadish, Cook, and Campbell [135] summarized John Stuart Mill’s three criteria for inferring causality: “A causal relationship exists if (1) the cause preceded the effect, (2) the cause was related to the effect, and (3) we can find no plausible alternative explanation for the effect other than the cause” (p. 6). Thus, the clearest evidence for a causal relationship comes from experimental rather than correlational research. From a strictly methodological standpoint, none of the studies included in our meta-analysis, not even those that used prospective measures of CM, such as child protection data, fulfill all of the three criteria. Potentially confounding variables were not included in most studies. A majority of the studies used a cross-sectional design in which psychopathic offenders retrospectively reported more childhood abuse than nonpsychopathic offenders. We cannot rule out the alternative hypothesis, that psychopathy caused CM because there is a possibility that psychopathic traits may have caused these individuals to retrospectively report higher rates of CM. The latter effect is generally referred to as ‘recall bias’ [136]. It occurs when the accuracy and inaccuracy in reporting prior experiences vary as a function of present (physical or psychological) health conditions. “One process believed to underlie the differential reporting accuracy is ‘effort after meaning,’ where unhealthy individuals exert more effort to search for disease explanation and assign more meaning to past events” [136]. Thus, people with psychopathic traits could attribute their problems in life to CM.

Second, the operationalization of CM varied greatly from study to study. In addition to different assessment measures, researchers also used diverse definitions of different types of CM. It would greatly benefit this field of study if scholars would agree on the operational definitions of the different types of CM. Perhaps, the definition of CM by the World Health Organization [137] could serve as a starting point. The 1999 WHO Consultation Group on Child Abuse Prevention distinguish four types of CM: physical abuse, emotional (or psychological) abuse, neglect, and sexual abuse. Recently, exposure to domestic violence has also been recognized as a separate form of CM by the WHO [137]. Additionally, the context of CM should be clearly operationalized in future studies because present day theories [e.g., 35] clearly distinguish between the psychological consequences of (chronic) maltreatment by primary caregivers versus other maltreatment of children (e.g., a single incident of sexual abuse by a stranger).

Third, although we managed to retrieve studies from six continents, samples from Western countries were clearly overrepresented in our meta-analysis. This has very likely impacted the findings, because the two variables we studied, psychopathy and CM, are not impervious to ethnic and cultural factors. A recent review of cultural and ethnic variations in psychopathy [138] concluded that there is evidence for ethnic differences in total psychopathy and facet scores, for instance, between African American and European American prisoners, as well as differential responding in laboratory tasks of emotional and cognitive processing between these groups. Thus, ethnic differences may exist in the way psychopathy manifests itself and its underlying mechanisms, possibly including its etiology. Furthermore, what is considered CM varies according to socially accepted norms, which are heavily influenced by culture. Certain parenting styles, such as corporal punishment, are viewed as inappropriate in some cultures, but are accepted and even promoted in others [139, 140]. Additionally, opinions about what constitutes child abuse have been found to differ amongst cultures (e.g., [141–143]). How cultural and ethnic factors shape the relationship between CM and psychopathic traits warrants further study.

Fourth, it should be noted that our results on the psychopathy factors and facets are limited to psychopathic traits as defined by Hare’s PCL-scales. Analyses on other psychopathy models could not be conducted due to a paucity of studies. Future research needs to examine how more novel models of psychopathy, such as the triarchic model, relate to CM [144–146].

Finally, some of our summary effect sizes, while statistically significant, are small in magnitude (i.e., sexual abuse). Small effects are to be expected for complex psychological constructs such as psychopathy and are not necessarily a limitation of this meta-analysis [95]. However, the issue of how to estimate the value of effect sizes is long debated, and scholars such as Robert Rosenthal [147] have convincingly argued that although the effect size is mathematically determined by characteristics of the study design and results, the interpretation of its real-life implications would depend upon the context (e.g., [148]) and the nature of the dependent variable. If childhood maltreatment is related to the development of psychopathic traits, even to a small extent, this is highly relevant information. The real-world implications of our findings are that prevention of childhood maltreatment could lead to a reduction of psychopathic traits, which in turn would lead to a reduction in antisocial and violent behavior in society at large.

## Conclusions

The current meta-analysis reports small to moderate effect sizes between psychopathy and CM. Effects are stronger for the behavioral lifestyle and antisocial facets than for the affective and interpersonal facets. We found that the associations of psychopathic traits with CM were largely invariant across gender, type of psychopathy measure, and publication type. The sample type was found to be a moderating factor.

Our findings support theoretical models and empirical research that suggest a role of childhood trauma, and CM by primary caregivers in particular, in the etiology of psychopathy. This role may be somewhat larger in the behavioral lifestyle (or emotional dysregulation) and the antisocial traits of psychopathy than in the affective (or emotional numbing) and interpersonal (narcissistic) symptoms, but still relevant to all components. Future research in this domain needs to focus on prospective, longitudinal designs across extensive time spans, because retrospective, cross-sectional designs cannot inform us about causal directions. Furthermore, a common set of CM measures, including behavioral observations would facilitate cross-study comparisons and future meta-analyses. Finally, experimental studies of the “trauma-psychopathy hypothesis”, such as evaluations of trauma-informed therapeutic interventions with children, adolescents, and adults with psychopathic traits will provide further insight into the mechanisms underlying this complex disorder.

## Supporting information

S1 Table Study characteristics of all included papers. % women = proportion of women in sample; ACEC = Adverse Childhood Experience Scale; AEQ = Abusive Experience Questionnaire; CASI = Comprehensive Adolescent Severity Index; CATS = Childhood Abuse and Trauma Scale; CECA = Childhood Experience of Care and Abuse; CMI-SF = Childhood Maltreatment Interview Schedule–Short Form; CPANS = Child Psychology Abuse and Neglect Scale; CTQ = Childhood Trauma Questionnaire; CTQ-SF = Childhood Trauma Questionnaire–Short Form; CTS = Conflict Tactics Scale; DD = Dirty Dozen; ETI = Early Trauma Inventory; FHHQ = Family Health History Questionnaire; LES = Life Events Scale; LSRP = Levenson Self-Report Psychopathy Scale; MASA = Multidimensional Assessment of Sex and Aggression; N = total number of participants; PCL-R = Psychopathy Checklist–Revised; PCL-SV = Psychopathy Checklist–Screening Version; PCL:YV = Psychopathy Checklist–Youth Version; PPI = Psychopathy Personality Inventory; PPI-R = Psychopathy Personality. ^a^ Cut-off: 0–19: low; 20–29: medium; ≥ 30: high. ^b^ Cut-off: 25. ^c^ Cut-off: 30. ^d^ Cut-off: 23. ^e^ Cut-off: 0–1: low; 2–9: medium; > 9: high.
(DOCX)

S2 Table Results of a multi-level meta-analysis, where all effect sizes are included in one model. *k* = number of effect sizes; *r* = pooled correlation; SE_*r*_ = standard error of *r*; CI = confidence interval of *r*; *σ*_1_^2^ = between-study heterogeneity; *σ*_2_^2^ = between-effect-size-within-study heterogeneity. ***p < .001.
(DOCX)

S1 File References for studies included in the meta-analysis. (DOCX)

S2 File PRISMA checklist. (PDF)

S1 Fig Forest plot showing effect sizes for general maltreatment. (EPS)

S2 Fig Forest plot showing effect sizes for physical abuse. (EPS)

S3 Fig Forest plot showing effect sizes for emotional abuse. (EPS)

S4 Fig Forest plot showing effect sizes for sexual abuse. (EPS)

S5 Fig Forest plot showing effect sizes for neglect. (EPS)

S6 Fig Funnel plots by type of childhood maltreatment assessing the presence of publication bias. (EPS)
