# Supplementary material for: A meta-analysis of childhood maltreatment in relation to psychopathic traits
Source: PLoS One. 2022 Aug 10;17(8):e0272704. doi: 10.1371/journal.pone.0272704 (PMC9365173; doi:10.1371/journal.pone.0272704)
Supplement: S1 Table — % women = proportion of women in sample; ACEC = Adverse Childhood Experience Scale; AEQ = Abusive Experience Questionnaire; CASI = Comprehensive Adolescent Severity Index; CATS = Childhood Abuse and Trauma Scale; CECA = Childhood Experience of Care and Abuse; CMI-SF = Childhood Maltreatment Interview Schedule–Short Form; CPANS = Child Psychology Abuse and Neglect Scale; CTQ = Childhood Trauma Questionnaire; CTQ-SF = Childhood Trauma Questionnaire–Short Form; CTS = Conflict Tactics Scale; DD = Dirty Dozen; ETI = Early Trauma Inventory; FHHQ = Family Health History Questionnaire; LES = Life Events Scale; LSRP = Levenson Self-Report Psychopathy Scale; MASA = Multidimensional Assessment of Sex and Aggression; N = total number of participants; PCL-R = Psychopathy Checklist–Revised; PCL-SV = Psychopathy Checklist–Screening Version; PCL:YV = Psychopathy Checklist–Youth Version; PPI = Psychopathy Personality Inventory; PPI-R = Psychopathy Personality. a Cut-off: 0–19: low; 20–29: medium; ≥ 30: high. b Cut-off: 25. c Cut-off: 30. d Cut-off: 23. e Cut-off: 0–1: low; 2–9: medium; > 9: high. (DOCX) [file pone.0272704.s001.docx]

|  |  |  |  |  |  | Measure | |
| --- | --- | --- | --- | --- | --- | --- | --- |
| Article | N | % women | Country | Sample type | Publication type | Psychopathy | Childhood maltreatment |
| Blonigen et al. (2012) | 215 | 100 | USA | Correctional | Journal article | PCL-R | PTE |
| Boduszek et al. (2019) | 325 | 100 | Barbados & Grenada | Community | Journal article | PPTS | Self-made questionnaire |
| Bohle & de Vogel (2017) | 418 | 51 | NL | Correctional | Journal article | PCL-R | Questionnaire to assess victimization |
| Borja & Ostrosky (2013) | 194 | 0 | Mexico | Correctional | Journal article | PCL-R^a^ | ETI |
| Campbell et al. (2004) | 226 | 17 | Canada | Correctional | Journal article | PCL:YV | AEQ |
| Cima et al. (2008) | 47 | 0 | NL | Correctional | Journal article | PPI | CTQ |
| Christopher et al. (2007) | 142 | 100 | USA | Correctional | Journal article | LSRP | CTQ |
| Cooke et al. (2020) | 789 | 62 | USA | Community | Journal article | LSRP | CMIS-SF/  Self-made questionnaire |
| Craparo et al. (2013) | 22 | 0 | Italy | Correctional | Journal article | PCL-R^b^ | TEC |
| Dargis et al. (2016) | 183 | 0 | USA | Correctional | Journal article | PCL-R | CTQ |
| Dargis & Koenigs (2018) | 222 | 0 | USA | Correctional | Journal article | PCL-R^c^ | CTQ |
| Durand & de Calheiros Velozo (2018) | 400 | 70 | USA | Community | Journal article | TriPM | CTQ-SF |
| Farina et al. (2018); Pennsylvania | 253 | 40 | USA | Correctional | Journal article | YPI | CTQ |
| Fisher (2003) | 110 | 23 | USA | Correctional | Dissertation | PSD | Official records |
| Forouzan & Nicholls (2015) | 32 | 100 | Canada | Community | Journal article | PCL-R^b^ | Purpose-built protocol |
| Gao et al. (2010) | 333 | 39 | Mauritius | Community | Journal article | SRP-II | CTS |
| Gao et al. (2011) | 71 | 0 | USA | Community | Journal article | PCL-R^d^ | CTS |
| Gowin et al. (2013) | 67 | 15 | USA | Clinical/Correctional | Journal article | SRP-III | CTQ |
| Grady et al. (2019) | 105 | 0 | Canada | Correctional | Journal article | PCL-R | Official records/self-report |
| Graham et al. (2012) | 223 | 0 | USA | Correctional | Journal article | PCL-R | Official records |
| Hong & Lishner (2016) | 248 | – | USA | Community | Journal article | SRP-III | SLEQ |
| Jia et al. (2020) | 991 | 76 | China | Community | Journal article | DD | CPANS |
| Kimonis et al. (2012) | 373 | 0 | USA | Correctional | Journal article | YPI | LES |
| Kolla et al. (2014) | 24 | 0 | UK | Correctional | Journal article | PCL-R^b^ | ETI |
| Koivisto & Haapasalo (1996) | 52 | 15 | Finland | Correctional | Journal article | PCL | Official records |
| Krischer & Sevecke (2008) | 185 | 48 | Germany | Correctional | Journal article | PCL:YV | CTQ |
| Krstic et al. (2016) | 397 | 0 | USA | Correctional | Journal article | PCL-R | MASA |
| Lang et al. (2002) | 199 | 0 | Sweden | Correctional | Journal article | PCL^e^ (shortened) | Official records/self-report |
| Marshall & Cooke (1999) | 105 | 0 | UK | Correctional | Journal article | PCL-R^b^ | CECA |
| McBride (1998); study 1 | 233 | 0 | Canada | Correctional | Dissertation | PCL:YV | Official records |
| Moore (2004) | 67 | 0 | USA | Correctional | Dissertation | PCL:YV | Official records |
| O’Neill et al. (2003) | 51 | 0 | USA | Clinical/Correctional | Journal article | PCL:YV | CASI |
| Ometto et al. (2016) | 107 | 44 | Brazil | Community | Journal article | PCL:YV | CTQ |
| Poythress et al. (2006) | 615 | 0 | USA | Correctional | Journal article | PCL-R | CATS |
| Rock (2016) | 220 | 35 | USA | Correctional | Dissertation | PCL-SV | FHHQ |
| Rose et al. (2020) | 68 | 0 | USA | Correctional | Journal article | PCL:YV | Self-report |
| Schimmenti et al. (2015) | 78 | 0 | Italy | Correctional | Journal article | PCL-R | TEC |
| Schraft et al. (2013) | 147 | 14 | USA | Correctional | Journal article | PCL:YV | CTQ-SF |
| Sevecke et al. (2016) | 334 | 50 | Germany | Correctional | Journal article | PCL:YV | CTQ |
| Strand et al. (2016) | 80 | 50 | Australia | Correctional | Journal article | PCL:YV | CTQ |
| Swogger et al. (2012) | 75 | 0 | USA | Correctional | Journal article | PCL-R | ACEC |
| Vahl et al. (2016) | 439 | 0 | NL | Correctional | Journal article | YPI | CTQ-SF |
| Verona et al. (2005) | 226 | 100 | USA | Correctional | Journal article | PCL-R | Official records/self-report |
| Waller et al. (2018) | 261 | 58 | USA | Community | Journal article | SRP-SF-IV | CTQ |
| Watts et al. (2017) | 1,169 | 73 | USA | Community | Journal article | PPI-R & LSRP | CTQ |
| Weiler & Widom (1996) | 1,069 | 50 | USA | Community | Journal article | PCL-R | Official records |
| Young & Widom (2014) | 547 | 58 | USA | Community | Journal article | PCL-R | Official records |
