# Supplementary material for: A meta-analysis of childhood maltreatment in relation to psychopathic traits
Source: PLoS One. 2022 Aug 10;17(8):e0272704. doi: 10.1371/journal.pone.0272704 (PMC9365173; doi:10.1371/journal.pone.0272704)
Supplement: S2 Table — k = number of effect sizes; r = pooled correlation; SEr = standard error of r; CI = confidence interval of r; σ12 = between-study heterogeneity; σ22 = between-effect-size-within-study heterogeneity. ***p < .001. (DOCX) [file pone.0272704.s002.docx]

|  | *k* | *r* | SE*_r_* | 95% CI | *σ*_1_^2^ | σ_2_^2^ |
| --- | --- | --- | --- | --- | --- | --- |
| Full multi-level model | 54 | .17^***^ | .01 | [.14, .19] | 0.0041 | 00053 |
| - |  |  |  |  |  |  |
| Subgroup analysis |  |  |  |  |  |  |
| --General maltreatment | 32 | .20^***^ | .02 | [.16, .24] |  |  |
| --Physical abuse | 32 | .19^***^ | .02 | [.15, .23] |  |  |
| --Emotional abuse | 25 | .15^***^ | .02 | [.11, .20] |  |  |
| --Sexual abuse | 32 | .10^***^ | .02 | [.06, .14] |  |  |
| --Neglect | 20 | .21^***^ | .03 | [.16, .26] |  |  |
