# Supplementary material for: A meta-analysis of childhood maltreatment in relation to psychopathic traits
Source: PLoS One. 2022 Aug 10;17(8):e0272704. doi: 10.1371/journal.pone.0272704 (PMC9365173; doi:10.1371/journal.pone.0272704)
Supplement: S1 File — (DOCX) [file pone.0272704.s003.docx]

Blonigen, D. M., Sullivan, E. A., Hicks, B. M., & Patrick, C. J. (2012). Facets of psychopathy in relation to potentially traumatic events and posttraumatic stress disorder among female prisoners: The mediating role of borderline personality disorder traits. *Personality Disorders: Theory, Research, and Treatment*, *3*(4), 406–414. https://doi.org/10.1037/a0026184

Boduszek, D., Debowska, A., Willmott, D., Jones, A. D., DeLisi, M., & Kirkman, G. (2019). Is female psychopathy linked with child abuse? An empirical investigation using a person-centered approach. *Journal of Child Sexual Abuse*, *28*(6), 708–725. https://doi.org/10.1080/10538712.2019.1592272

Bohle, A., & de Vogel, V. (2017). Gender differences in victimization and the relation to personality disorders in forensic psychiatry. *Journal of Aggression, Maltreatment & Trauma*, *26*(4), 411–429. https://doi.org/10.1080/10926771.2017.1284170

Borja, K., & Ostrosky, F. (2013). Early traumatic events in psychopaths. *Journal of Forensic Sciences*, *58*(4), 927–931. https://doi.org/10.1111/1556-4029.12104

Campbell, M. A., Porter, S., & Santor, D. (2004). Psychopathic traits in adolescent offenders: An evaluation of criminal history, clinical, and psychosocial correlates. *Behavioral Sciences & the Law*, *22*(1), 23–47. https://doi.org/10.1002/bsl.572

Christopher, K., Lutz-Zois, C. J., & Reinhardt, A. R. (2007). Female sexual-offenders: Personality pathology as a mediator of the relationship between childhood sexual abuse history and sexual abuse perpetration against others. *Child Abuse & Neglect*, *31*(8), 871–883. https://doi.org/10.1016/j.chiabu.2007.02.006

Cima, M., Smeets, T., & Jelicic, M. (2008). Self-reported trauma, cortisol levels, and aggression in psychopathic and non-psychopathic prison inmates. *Biological Psychology*, *78*(1), 75–86. https://doi.org/10.1016/j.biopsycho.2007.12.011

Cooke, E. M., Lewis, R. H., Hayes, B. E., Bouffard, L. A., Boisvert, D. L., Wells, J., Kavish, N., Woeckener, M., & Armstrong, T. A. (2020). Examining the relationship between victimization, psychopathy, and the acceptance of rape myths. *Journal of Interpersonal Violence*, 1–21. https://doi.org/10.1177/0886260520966669

Craparo, G., Schimmenti, A., & Caretti, V. (2013). Traumatic experiences in childhood and psychopathy: A study on a sample of violent offenders from Italy. *European Journal of Psychotraumatology*, *4*(1). https://doi.org/10.3402/ejpt.v4i0.21471

Dargis, M., & Koenigs, M. (2018). Two subtypes of psychopathic criminals differ in negative affect and history of childhood abuse. *Psychological Trauma: Theory, Research, Practice, and Policy*, *10*(4), 444–451. https://doi.org/10.1037/tra0000328

Dargis, M., Newman, J., & Koenigs, M. (2016). Clarifying the link between childhood abuse history and psychopathic traits in adult criminal offenders. *Personality Disorders: Theory, Research, and Treatment*, *7*(3), 221–228. https://doi.org/10.1037/per0000147

Durand, G., & de Calheiros Velozo, J. (2018). The interplay of gender, parental behaviors, and child maltreatment in relation to psychopathic traits. *Child Abuse*, *83*, 120–128. https://doi.org/10.1016/j.chiabu.2018.07.013

Farina, A. S. J., Holzer, K. J., DeLisi, M., & Vaughn, M. G. (2018). Childhood trauma and psychopathic features among juvenile offenders. *International Journal of Offender Therapy and Comparative Criminology*, *62*(14), 4359–4380. https://doi.org/10.1177/0306624X18766491

Fisher, A. G. (2003). *The relationship of psychopathy and abuse victimization to level of juvenile sexually problematic behavior* (Publication No. 3115926) [Doctoral dissertation, DePaul University]. ProQuest Dissertations Publishing.

Forouzan, E., & Nicholls, T. L. (2015). Childhood and adolescent characteristics of women with high versus low psychopathy scores: Examining developmental precursors to the malignant personality disorder. *Journal of Criminal Justice*, *43*(4), 307–320. https://doi.org/10.1016/j.jcrimjus.2015.06.001

Gao, Yu, Raine, A., Chan, F., Venables, P. H., & Mednick, S. A. (2010). Early maternal and paternal bonding, childhood physical abuse and adult psychopathic personality. *Psychological Medicine*, *40*(6), 1007–1016. https://doi.org/10.1017/S0033291709991279

Gao, Yu, Raine, A., & Schug, R. A. (2011). P3 event-related potentials and childhood maltreatment in successful and unsuccessful psychopaths. *Brain and Cognition*, *77*(2), 176–182. https://doi.org/10.1016/j.bandc.2011.06.010

Gowin, J. L., Green, C. E., Alcorn, J. L., Swann, A. C., Moeller, F. G., & Lane, S. D. (2013). The role of cortisol and psychopathy in the cycle of violence. *Psychopharmacology*, *227*(4), 661–672. https://doi.org/10.1007/s00213-013-2992-1

Grady, M. D., Looman, J., & Abracen, J. (2019). Childhood abuse, attachment, and psychopathy among individuals who commit sexual offenses. *Sexual Addiction & Compulsivity*, *26*(1–2), 77–102. https://doi.org/10.1080/10720162.2019.1620660

Graham, N., Kimonis, E. R., Wasserman, A. L., & Kline, S. M. (2012). Associations among childhood abuse and psychopathy facets in male sexual offenders. *Personality Disorders: Theory, Research, and Treatment*, *3*(1), 66–75. https://doi.org/10.1037/a0025605

Hong, P. Y., & Lishner, D. A. (2016). General invalidation and trauma-specific invalidation as predictors of personality and subclinical psychopathology. *Personality and Individual Differences*, *89*, 211–216. https://doi.org/10.1016/j.paid.2015.10.016

Jia, X., Wang, Q., & Lin, L. (2020). The relationship between childhood neglect and malevolent creativity: The mediating effect of the dark triad personality. *Frontiers in Psychology*, *11*, 613695. https://doi.org/10.3389/fpsyg.2020.613695

Kimonis, E. R., Frick, P. J., Cauffman, E., Goldweber, A., & Skeem, J. (2012). Primary and secondary variants of juvenile psychopathy differ in emotional processing. *Development and Psychopathology*, *24*(3), 1091–1103. https://doi.org/10.1017/S0954579412000557

Koivisto, H., & Haapasalo, J. (1996). Childhood maltreatment and adulthood psychopathy in light of file-based assessments among mental state examinees. *Studies on Crime & Crime Prevention*, *5*(1), 91–104.

Kolla, N. J., Gregory, S., Attard, S., Blackwood, N., & Hodgins, S. (2014). Disentangling possible effects of childhood physical abuse on gray matter changes in violent offenders with psychopathy. *Psychiatry Research: Neuroimaging*, *221*(2), 123–126. https://doi.org/10.1016/j.pscychresns.2013.11.008

Krischer, M. K., & Sevecke, K. (2008). Early traumatization and psychopathy in female and male juvenile offenders. *International Journal of Law and Psychiatry*, *31*(3), 253–262. https://doi.org/10.1016/j.ijlp.2008.04.008

Krstic, S., Knight, R. A., & Robertson, C. A. (2016). Developmental antecedents of the facets of psychopathy: The role of multiple abuse experiences. *Journal of Personality Disorders*, *30*(5), 677–693. https://doi.org/10.1521/pedi_2015_29_223

Lang, S., af Klinteberg, B., & Alm, P.-O. (2002). Adult psychopathy and violent behavior in males with early neglect and abuse. *Acta Psychiatrica Scandinavica*, *106*(s412), 93–100. https://doi.org/10.1034/j.1600-0447.106.s412.20.x

Marshall, L. A., & Cooke, D. J. (1999). The childhood experiences of psychopaths: A retrospective study of familial and societal factors. *Journal of Personality Disorders*, *13*(3), 211–225. https://doi.org/10.1521/pedi.1999.13.3.211

McBride, M. L. (1998). *Individual and familial risk factors for adolescent psychopathy [Doctoral dissertation, University of British Columbia]*. University of British Columbia Library. http://hdl.handle.net/2429/9575

Moore, R. L. (2004). *The association between psychopathy and autonomic reactivity in young adolescents* (Publication No. 3155833) [Doctoral dissertation, Alliant International University]. ProQuest Dissertations Publishing.

Ometto, M., de Oliveira, P. A., Milioni, A. L., dos Santos, B., Scivoletto, S., Busatto, G. F., Nunes, P. V., & Cunha, P. J. (2016). Social skills and psychopathic traits in maltreated adolescents. *European Child & Adolescent Psychiatry*, *25*(4), 397–405. https://doi.org/10.1007/s00787-015-0744-y

O’Neill, M. L., Lidz, V., & Heilbrun, K. (2003). Predictors and correlates of psychopathic characteristics in substance abusing adolescents. *International Journal of Forensic Mental Health*, *2*(1), 35–45. https://doi.org/10.1080/14999013.2003.10471177

Poythress, N. G., Skeem, J. L., & Lilienfeld, S. O. (2006). Associations among early abuse, dissociation, and psychopathy in an offender sample. *Journal of Abnormal Psychology*, *115*(2), 288–297. https://doi.org/10.1037/0021-843X.115.2.288

Rock, R. C. (2016). *An examination of the relationship between adult psychopathy and childhood trauma in a jail sample* [Doctoral dissertation, The University of Alabama]. University of Alabama Libraries. https://ir.ua.edu/handle/123456789/2813

Rose, K., Woodworth, D. M., & Minton, J. (2020). An exploration of individual differences in a sample of youth charged with violent sexual and non-sexual crimes. *Psychiatry, Psychology and Law*, 1–18. https://doi.org/10.1080/13218719.2019.1687043

Schimmenti, A., Di Carlo, G., Passanisi, A., & Caretti, V. (2015). Abuse in childhood and psychopathic traits in a sample of violent offenders. *Psychological Trauma: Theory, Research, Practice, and Policy*, *7*(4), 340–347. https://doi.org/10.1037/tra0000023

Schraft, C. V., Kosson, D. S., & McBride, C. K. (2013). Exposure to violence within home and community environments and psychopathic tendencies in detained adolescents. *Criminal Justice and Behavior*, *40*(9), 1027–1043. https://doi.org/10.1177/0093854813486887

Sevecke, K., Franke, S., Kosson, D., & Krischer, M. (2016). Emotional dysregulation and trauma predicting psychopathy dimensions in female and male juvenile offenders. *Child and Adolescent Psychiatry and Mental Health*, *10*(1), 43. https://doi.org/10.1186/s13034-016-0130-7

Strand, S., Luebbers, S., & Shepherd, S. M. (2016). Psychopathic features in young incarcerated females. *Journal of Criminal Psychology*, *6*(2), 63–75. https://doi.org/10.1108/JCP-02-2016-0004

Swogger, M. T., Walsh, Z., Kosson, D. S., Cashman-Brown, S., & Caine, E. D. (2012). Self-reported childhood physical abuse and perpetration of intimate partner violence: The moderating role of psychopathic traits. *Criminal Justice and Behavior*, *39*(7), 910–922. https://doi.org/10.1177/0093854812438160

Vahl, P., Colins, O. F., Lodewijks, H. P. B., Lindauer, R., Markus, M. T., Doreleijers, T. A. H., & Vermeiren, R. R. (2016). Psychopathic traits and maltreatment: Relations with aggression and mental health problems in detained boys. *International Journal of Law and Psychiatry*, *46*, 129–136. https://doi.org/10.1016/j.ijlp.2016.02.006

Verona, E., Hicks, B. M., & Patrick, C. J. (2005). Psychopathy and suicidality in female offenders: Mediating influences of personality and abuse. *Journal of Consulting and Clinical Psychology*, *73*(6), 1065–1073. https://doi.org/10.1037/0022-006X.73.6.1065

Waller, R., McCabe, H. K., Dotterer, H. L., Neumann, C. S., & Hyde, L. W. (2018). Unique and interactive associations between maltreatment and complex emotion recognition deficits and psychopathic traits in an undergraduate sample. *Journal of Personality Disorders*, *32*(4), 543–561. https://doi.org/10.1521/pedi_2017_31_314

Watts, A. L., Donahue, K., Lilienfeld, S. O., & Latzman, R. D. (2017). Gender moderates psychopathic traits’ relations with self-reported childhood maltreatment. *Personality and Individual Differences*, *119*, 175–180. https://doi.org/10.1016/j.paid.2017.07.011

Weiler, B. L., & Widom, C. S. (1996). Psychopathy and violent behaviour in abused and neglected young adults. *Criminal Behaviour and Mental Health*, *6*(3), 253–271. https://doi.org/10.1002/cbm.99

Young, J. C., & Widom, C. S. (2014). Long-term effects of child abuse and neglect on emotion processing in adulthood. *Child Abuse & Neglect*, *38*(8), 1369–1381. https://doi.org/10.1016/j.chiabu.2014.03.008
